# Association between base excess level at hospital arrival and neurological outcomes in adult out-of-hospital cardiac arrest: A multicentre cohort study

**DOI:** 10.1016/j.resplu.2025.101055

**Published:** 2025-08-06

**Authors:** Ryuta Onodera, Norihiro Nishioka, Tomoki Yamada, Shunichiro Nakao, Kazuhisa Yoshiya, Changhwi Park, Tetsuro Nishimura, Takuya Ishibe, Kazuma Yamakawa, Takeyuki Kiguchi, Masafumi Kishimoto, Kohei Ninomiya, Yusuke Ito, Taku Sogabe, Takaya Morooka, Haruko Sakamoto, Yuki Hironaka, Atsunori Onoe, Tasuku Matsuyama, Yohei Okada, Satoshi Matsui, Satoshi Yoshimura, Shunsuke Kimata, Shunsuke Kawai, Yuto Makino, Ling Zha, Kosuke Kiyohara, Tetsuhisa Kitamura, Taku Iwami, CRITICAL Study Group Investigators

**Affiliations:** a Department of Preventive Services, Kyoto University School of Public Health, Kyoto, Japan; b Emergency and Critical Care Medical Center, Osaka Police Hospital, Osaka, Japan; c Department of Traumatology and Acute Critical Medicine, Graduate School of Medicine, The University of Osaka, Suita, Japan; d Department of Emergency and Critical Care Medicine, Kansai Medical University, Takii Hospital, Moriguchi, Japan; e Department of Emergency Medicine, Tane General Hospital, Osaka, Japan; f Department of Traumatology and Critical Care Medicine, Osaka Metropolitan University, Osaka, Japan; g Department of Emergency and Critical Care Medicine, Kindai University School of Medicine, Osaka-Sayama, Japan; h Department of Emergency and Critical Care Medicine Osaka Medical and Pharmaceutical University Takatsuki Japan; i Critical Care and Trauma Center, Osaka General Medical Center, Osaka, Japan; j Osaka Prefectural Nakakawachi Medical Center of Acute Medicine, Higashi-Osaka, Japan; k Senshu Trauma and Critical Care Center, Osaka, Japan; l Senri Critical Care Medical Center, Saiseikai Senri Hospital, Suita, Japan; m Traumatology and Critical Care Medical Center, National Hospital Organization Osaka National Hospital, Osaka, Japan; n Emergency and Critical Care Medical Center, Osaka City General Hospital, Osaka, Japan; o Department of Pediatrics, Osaka Red Cross Hospital, Osaka, Japan; p Emergency and Critical Care Medical Center, Kishiwada Tokushukai Hospital, Osaka, Japan; q Department of Emergency and Critical Care Medicine, Kansai Medical University, Hirakata, Osaka, Japan; r Department of Emergency Medicine, Kyoto Prefectural University of Medicine, Kyoto, Japan; s Division of Emergency Medicine, Hyogo Prefectural Kobe Children’s Hospital, Kobe, Japan; t Department of Anesthesiology and Intensive Care Medicine, Nagoya City University Graduate School of Medical Sciences, Nagoya, Japan; u Division of Environmental Medicine and Population Sciences, Department of Social and Environmental Medicine, Graduate School of Medicine, The University of Osaka, Suita, Japan; v Department of Food Science, Otsuma Women’s University, Tokyo, Japan

**Keywords:** Base excess, BE, Blood gas, Out-of-hospital cardiac arrest

## Abstract

**Aim:**

We aimed to assess the association between base excess (BE) levels and neurological outcomes in patients with out-of-hospital cardiac arrest (OHCA), accounting for the time from cardiac arrest onset to blood sampling.

**Methods:**

This multicentre study was conducted in Osaka, Japan, and enrolled consecutive patients with OHCA who were transported to 16 medical centres between 2012 and 2021. Patients aged ≥ 18 years with witnessed OHCA and available BE measurements upon hospital arrival were examined. Patients were stratified into Q1 (BE ≤ −21.1 mmol/L), Q2 (−21.1 < BE ≤ −15.7 mmol/L), Q3 (−15.7 < BE ≤ −10.4 mmol/L) and Q4 (BE > −10.4 mmol/L) groups based on BE levels. The primary outcome was 1-month survival with a favourable neurological outcome (Cerebral Performance Category scale score: 1 or 2).

**Results:**

Among the 23,854 patients with OHCA, only 6066 were included in the final analysis. Approximately 3.2 %, 4.7 %, 9.9 % and 23.7 % of patients in the Q1, Q2, Q3 and Q4 groups, respectively, achieved favourable neurological outcomes at 1 month. Compared with Q4, the adjusted odds ratio for a favourable neurological outcome in Q1 was 0.13. Subgroup analysis revealed a significant interaction between prehospital return of spontaneous circulation (ROSC) and neurological outcomes; neurological outcomes worsened as BE decreased in patients with ROSC but not in those without ROSC.

**Conclusion:**

Lower BE levels upon hospital arrival are associated with poorer neurological outcomes and may serve as prognostic indicators in patients with OHCA who achieved prehospital ROSC.

## Highlights

• Lower BE levels were associated with worse neurological outcomes after OHCA.
• BE levels were categorised into quartiles to assess outcome associations.
• Approximately 3.2% (Q1)–23.7% (Q4) of patients showed favourable 1-month outcomes.
• BE showed stronger prognostic value in patients with prehospital ROSC.
• BE may serve as an early prognostic indicator in patients with ROSC.

## Introduction

Out-of-hospital cardiac arrest (OHCA) remains a significant global public health concern, with persistently low survival rates and frequently poor neurological outcomes.^1^ The management of OHCA is inherently complex, requiring multidisciplinary interventions, prolonged treatment, and substantial medical and human resources. Despite the successful return of spontaneous circulation (ROSC), many patients subsequently experience mortality or progress to unresponsive wakefulness syndrome, even after receiving post-resuscitation care.^2^, ^3^ Consequently, the early prediction of outcomes in patients with OHCA is critical. Accurate prognostication can facilitate the timely and appropriate implementation of intensive care interventions, such as extracorporeal membrane oxygenation (ECMO) and targeted temperature management (TTM), and guide decisions regarding the continuation or termination of resuscitative efforts.

Cardiac arrest results in the abrupt cessation of blood flow, leading to severe hypercarbia and metabolic acidosis at the tissue level.^4^ Metabolic acidosis exerts several deleterious physiological effects, including impaired myocardial function,^5^ peripheral vasodilation^6^ and suppression of inflammatory and immune responses,^7^ which can contribute to the development of multiple organ failure.

Although metabolic acidosis is commonly observed following cardiac arrest and often attributed to elevated lactate levels,^8^, ^9^ its underlying mechanisms are multifactorial. Notably, lactate levels account for only approximately 50 % of the metabolic acidosis observed during cardiac arrest.^10^

The standard base excess (BE) is a useful parameter for evaluating the underlying causes of metabolic acidosis.^10^ BE, introduced in 1960 as a metric used to quantify the severity of metabolic acidosis, is widely used in clinical practice.^11^, ^12^, ^13^, ^14^ It can be rapidly obtained through blood gas analysis. BE represents the amount of base, measured in millimoles, required to restore the pH of 1 L of fully oxygen-saturated arterial blood to 7.40 at a temperature of 37 °C and a partial pressure of carbon dioxide of 40 Torr. The normal BE range is between +2 and –2 mmol/L.^15^ Standard BE (SBE) has been widely investigated as a prognostic marker in trauma and acute care settings.^16^ In patients with cardiac arrest, a lower BE level is associated with poorer outcomes.^17^, ^18^, ^19^ However, previous studies were limited by small sample size and lack of adjustment for the time from cardiac arrest to blood sampling—an important confounding factor that reflects the progression of metabolic acidosis, including lactate accumulation. Without adjusting for this variable, the association between BE and neurological outcomes may be inaccurately estimated.

Furthermore, prehospital ROSC is an important clinical factor influencing a patient’s physiological status upon hospital arrival.^20^ Most previous studies have focused primarily on patients who achieved ROSC prior to hospital admission,^18^, ^19^, ^21^ leaving the prognostic significance of BE in those without ROSC largely unclear.

Therefore, this study aimed to assess the association between BE levels upon hospital arrival and neurological outcomes in patients with OHCA using data from a large-scale, multicentre registry in Osaka, Japan, where termination of resuscitation by emergency medical service (EMS) personnel is not permitted. The analysis accounted for the time from the onset of cardiac arrest to blood sampling. The potential modifying effect of prehospital ROSC on the association between BE levels and neurological outcomes was also examined.

## Methods

The study was approved by the Ethics Committee of Kyoto University and the institutional review boards of all participating institutions (R1045). The requirement for written informed consent was waived owing to the retrospective nature of the study.

### Study design and setting

The retrospective study used data from the Comprehensive Registry of Intensive Cares for OHCA Survival (CRITICAL) study. The CRITICAL study is a prospective, multicentre registry designed to collect and evaluate both prehospital and in-hospital treatments for OHCA. Prehospital data were obtained from the All-Japan Utstein Registry, maintained by the Fire and Disaster Management Agency (FDMA).^22^, ^23^, ^24^, ^25^ In-hospital data were collected from 15 tertiary critical care medical centres and 1 community hospital with an emergency department, all located in Osaka Prefecture, Japan. Osaka Prefecture is a highly urbanised region covering an area of 1905 km^2^, with an estimated population of approximately 8.8 million as of 2020. Approximately 7500 individuals experience OHCA annually in the region (FDMA of the Ministry of Internal Affairs and Communications website. Available from: https://www.fdma.go.jp/publication/rescue/post7.html). Of these, approximately one in four patients with OHCA (approximately ≥2000 per year) were enrolled in the CRITICAL registry from 2012 to 2021.

The registry remains active without a predefined end date. In-hospital data were documented by attending physicians and subsequently entered into a standardised online data collection form by either physicians or trained medical administrators. To ensure data quality, all entries were reviewed and verified by the CRITICAL study working group.^25^ Incomplete data were returned to the respective institutions for correction and completion. Further details regarding the All-Japan Utstein Registry and the CRITICAL study have been reported in a previous study.^25^

### Emergency medical services system in Osaka

The EMS system in Japan has been previously described in detail.^26^, ^27^ Emergency assistance is accessible nationwide by dialling 119, after which the nearest available ambulance is promptly dispatched by an emergency call centre. Services are available 24 h a day. Each ambulance is staffed by a three-member team trained in providing life support measures. The most qualified EMS personnel, known as emergency lifesaving technicians, are authorised to establish an intravenous access using lactated Ringer’s solution, administer adrenaline, secure an adjunctive airway, and operate a semiautomated external defibrillator (AED) in patients with OHCA. Technicians with advanced training are additionally permitted to perform tracheal intubation on such patients. However, the administration of sodium bicarbonate for the correction of acidosis is not permitted.

In Japan, EMS personnel are generally not authorised to comply with do-not-resuscitate orders. Consequently, resuscitation is typically initiated and continued at the scene, and nearly all patients with OHCA are transported to medical facilities. Exceptions are made only in the presence of unequivocal signs of death—such as decapitation, incineration, decomposition, rigor mortis or dependent cyanosis. All EMS providers performed cardiopulmonary resuscitation (CPR) in accordance with the Japanese CPR guidelines.^28^

### Study population

Patients aged 18 years with witnessed OHCA and whose BE levels were measured upon arrival at the hospital from 1 January 2012 to 31 December 2021 were enrolled in this study. Meanwhile, patients with OHCA due to external causes (e.g. trauma, hanging, drowning, drug overdose or asphyxia), with unknown measurement time, and in whom ECMO was initiated prior to the BE measurement were excluded.

### Exposures

The main exposure variable was the BE value obtained from the arterial blood gas analysis, which was conducted as part of the initial blood test performed upon hospital arrival. Patients were divided into quartiles based on BE values recorded at admission^29^: Q1 (BE ≤ −21.1 mmol/L), Q2 (−21.1 < BE ≤ −15.7 mmol/L), Q3 (−15.7 < BE ≤ −10.4 mmol/L) and Q4 (BE > −10.4 mmol/L) groups. In the present study, no standardised protocol was implemented to determine whether SBE or actual BE (ABE) should be used. Furthermore, the type of blood gas analyser used varied across participating institutions. These methodological decisions were made at the discretion of the attending physicians or in accordance with institutional policies.

### Outcomes

The primary outcome was 30-day survival with favourable neurological outcomes, defined as a Cerebral Performance Category (CPC) score of 1 or 2. Neurological status was evaluated by the treating physician using the CPC scale, which is categorised as follows: 1, good cerebral performance; 2, moderate cerebral disability; 3, severe cerebral disability; 4, coma or vegetative state; and 5, death or brain death.^30^ The secondary outcome was 1-month survival.

### Statistical analyses

Continuous variables were expressed as the medians with interquartile ranges, whereas categorical variables were expressed as proportions. The baseline characteristics across BE quartiles were compared using the Kruskal–Wallis test for continuous variables and the chi-square test for categorical variables. Multivariable logistic regression analysis was performed to estimate the adjusted odds ratios (AORs) with their corresponding 95 % confidence intervals (CIs) for the associations between BE quartiles and each outcome. Trends across BE quartiles were assessed using the Cochran–Armitage trend test. The primary regression model was adjusted for clinically relevant variables, including age, sex (male or female), cause of cardiac arrest (cardiac or non-cardiac), presence of bystander CPR, use of an AED by a bystander, initial documented rhythm (shockable, non-shockable or unknown) at the scene and in hospital, administration of adrenaline, advanced airway management, and defibrillation by EMS, no-flow time (defined as the time from the witnessed arrest to the initiation of CPR by a bystander or EMS personnel), time from EMS call to hospital arrival and time from hospital arrival to blood sampling.

The interpretation of BE values may be challenging in patients with preexisting kidney failure or chronic lung disease.^15^ Therefore, as a sensitivity analysis, arterial carbon dioxide pressure (PCO_2_), obtained from the same blood gas sample as the BE measurement, and creatinine levels upon hospital arrival were added to the primary regression model to assess the robustness of the findings. In a separate sensitivity analysis, low-flow time for CPR was included as an additional covariate, based on previous studies suggesting that BE values may be influenced by the duration of CPR.^18^ Low-flow time was defined as the interval from the initiation of CPR by bystanders or EMS personnel to the ROSC in patients who achieved ROSC before blood sampling, and as the interval from CPR initiation to blood sampling after hospital arrival in patients without ROSC. Additionally, SBE was recalculated using arterial pH and pCO_2_ values based on the following formula: SBE = (0.0307 × pCO_2_ × 10^(pH − 6.105)) − 24.8 + 16.2 × (pH − 7.40).^15^ The primary analyses were subsequently repeated using the recalculated SBE values to assess the consistency of the findings.

To illustrate the potential nonlinear relationship between BE levels and the probability of favourable neurological outcomes, a restricted cubic spline model was applied.^31^ Three knots were incorporated based on the assumption that the association between BE and neurological outcomes would be monotonic or only mildly nonlinear. A subgroup analysis was conducted to evaluate BE based on the presence or absence of prehospital ROSC. Moreover, the potential interaction between BE and other clinical variables on neurological and survival outcomes was assessed using a multivariate logistic regression model that included cross-product terms. All statistical analyses were performed using the R software (R Foundation for Statistical Computing version 4.2.1) and Stata software (version 17; StataCorp, College Station, TX, USA). A complete case analysis was conducted, as missing data for key variables were minimal following the application of the exclusion criteria (Supplementary Table 1). All tests were two-tailed, and a p-value of <0.05 was considered significant.

## Results

**Fig. 1:**
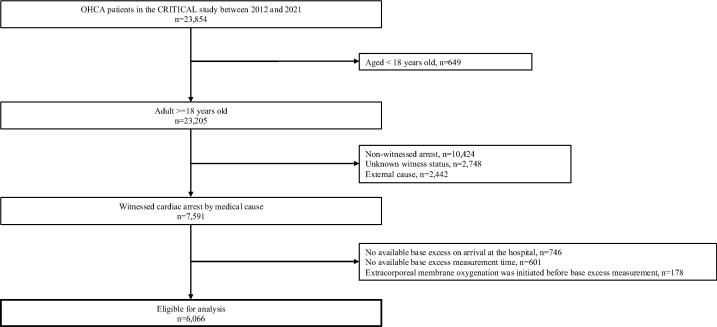
Flow diagram of patients with out-of-hospital cardiac arrest who had base excess values measured upon hospital arrival. Abbreviation: OHCA, out-of-hospital cardiac arrest.

A total of 23,854 patients with OHCA recorded in the CRITICAL study between 2012 and 2021 were screened (Fig. 1). Of these, 7591 patients were identified as having sustained witnessed cardiac arrest due to medical causes upon hospital arrival. After applying the eligibility criteria, only 6066 patients were included in the final analysis. These patients were stratified into the following quartiles based on BE values: Q1 (BE ≤ –21.1 mmol/L; n = 1528), Q2 (–21.1 < BE ≤ –15.7 mmol/L; n = 1520), Q3 (–15.7 < BE ≤ –10.4 mmol/L; n = 1513) and Q4 (BE > –10.4 mmol/L; n = 1505) groups.

The patient characteristics, pre- and in-hospital information and clinical outcomes of eligible patients are presented in Table 1. Compared with the other groups, patients in the Q1 group were less likely to experience a witnessed arrest, receive bystander CPR or bystander shock delivery by an AED, present with an initial shockable rhythm and undergo TTM. The proportion of patients with favourable neurological outcomes at 30 days was lowest in the Q1 group at 3.2 % (49/1528), followed by 4.7 % (72/1520) in the Q2 group, 9.9 % (149/1513) in the Q2 group and 23.7 % (356/1505) in the Q4 group (Table 2). A significant decreasing trend in favourable neurological outcomes was observed with lower BE values (p for trend <0.001, Table 2). The restricted cubic spline analysis demonstrated a nonlinear positive association between BE and the predicted probability of a favourable neurological outcome (p for nonlinearity <0.001) (Supplementary Fig. 1).

**Table 1:** Baseline characteristics of patients who experienced out-of-hospital cardiac arrest categorised based on base excess levels.

Variables	Total n = 6066	Base excess (BE), mmol/L				
		Quartile 1 (BE ≤ –21.1) n = 1528	Quartile 2 (–21.1 < BE ≤ −15.7) n = 1520	Quartile 3 (−15.7 < BE ≤ −10.4) n = 1513	Quartile 4 (BE > −10.4) n = 1505	p-value
*Prehospital characteristics*						
Age (years)	73 [63–82]	72 [59–80]	74 [64–82]	74 [64–82]	74 [64–82]	<0.001
Male (%)	3976 (65.6)	1050 (68.7)	975 (64.1)	1002 (66.2)	949 (63.1)	0.006
Cardiac cause (%)	4541 (74.9)	1137 (74.4)	1133 (74.5)	1139 (75.3)	1132 (75.2)	0.921
Type of cardiac cause (%)						<0.001
Acute coronary syndrome	722 (11.9)	135 (8.8)	183 (12.0)	173 (11.4)	231 (15.3)	
Other cardiac disease	673 (11.1)	99 (6.5)	134 (8.8)	192 (12.7)	248 (16.5)	
Probable cardiac disease	3146 (51.9)	903 (59.1)	816 (53.7)	774 (51.2)	653 (43.4)	
Layperson bystander CPR (%)	2399 (39.6)	539 (35.3)	600 (39.5)	609 (40.3)	651 (43.3)	<0.001
Type of bystander (%)						0.01
Lay person	4820 (79.5)	1176 (77.0)	1201 (79.0)	1212 (80.1)	1231 (81.8)	
EMS	1246 (20.5)	352 (23.0)	319 (21.0)	301 (19.9)	274 (18.2)	
Bystander shock delivery by an AED (%)	200 (3.3)	27 (1.8)	36 (2.4)	47 (3.1)	90 (6.0)	<0.001
Initial documented rhythm (%)						<0.001
Shockable	1224 (20.2)	214 (14.0)	284 (18.7)	334 (22.1)	392 (26.0)	
Non-shockable	4838 (79.8)	1312 (86.0)	1236 (81.3)	1178 (77.9)	1112 (73.9)	
Defibrillation by EMS (%)	1607 (26.5)	320 (20.9)	386 (25.4)	424 (28.0)	477 (31.7)	<0.001
Adrenaline administration by EMS (%)	1930 (31.8)	563 (36.9)	592 (38.9)	453 (29.9)	322 (21.4)	<0.001
Advanced airway management by EMS (%)	2934 (48.4)	793 (51.9)	813 (53.5)	724 (47.9)	604 (40.1)	<0.001
No-flow time for CPR (min)	3 [0–9]	3 [0–10]	3 [0–9]	3 [0–9]	3 [0–8]	0.264
Low-flow time for CPR (min)	27 [17–37]	31 [22–41]	29 [20–38]	26 [17–36]	21 [11–32]	<0.001
Time from witness to hospital arrival (min)	31 [22–39]	32 [23–41]	31 [23–40]	30 [22–38]	29 [22–37]	<0.001
Time from call to hospital arrival (min)	32 [27–39]	34 [28–41]	33 [27–40]	32 [26–38]	30 [25–37]	<0.001
*In-hospital characteristics*						
Initial documented rhythm (%)						<0.001
Shockable	468 (7.7)	112 (7.3)	133 (8.8)	121 (8.0)	102 (6.8)	
Non-shockable	4644 (76.6)	1323 (86.6)	1215 (79.9)	1160 (76.7)	946 (62.9)	
Presence of pulse	954 (15.7)	93 (6.1)	172 (11.3)	232 (15.3)	457 (30.4)	
Defibrillation in hospital (%)	943 (15.5)	259 (17.0)	266 (17.5)	246 (16.3)	172 (11.4)	<0.001
Time from hospital arrival to blood test (min)	7 [3–13]	8 [4–15]	7 [3–12]	7 [3–12]	6 [3–12]	<0.001
Coronary angiography (%)	1094 (18.0)	204 (13.4)	244 (16.1)	257 (17.0)	389 (25.8)	<0.001
Target temperature management (%)	774 (12.8)	120 (7.9)	182 (12.0)	210 (13.9)	262 (17.4)	<0.001
Extracorporeal membrane oxygenation initiated after blood test (%)	516 (8.5)	179 (11.7)	166 (10.9)	106 (7.0)	65 (4.3)	<0.001
Laboratory data						
pH	6.98 [6.85–7.11]	6.78 [6.70–6.85]	6.93 [6.87–7.00]	7.03 [6.96–7.11]	7.17 [7.08–7.29]	<0.001
Lactate (mmol/L)	11.8 [8.7–15.2]	17.0 [14.6–19.7]	13.0 [11.2–15.0]	10.6 [8.8–12.3]	7.4 [5.8–9.2]	<0.001
HCO_3–_ (mmol/L)	15.5 [11.6–19.5]	10.3 [7.6–12.8]	14.0 [11.5–16.4]	17.5 [14.6–19.8]	21.4 [18.6–23.9]	<0.001
PaCO_2_ (mmHg)	70.2 [50.6–89.9]	73.4 [54.0–97.8]	72.5 [52.8–92.6]	72.1 [52.9–89.4]	63.9 [44.2–80.1]	<0.001
Creatinine (mg/dL)	1.19 [0.92–1.65]	1.41 [1.10–2.20]	1.20 [0.95–1.60]	1.13 [0.90–1.60]	1.03 [0.83–1.30]	<0.001

Data are expressed as the medians [interquartile range, IQR] for continuous variables and n (%) for categorical variables.

Abbreviations: IQR, interquartile range; CPR, cardiopulmonary resuscitation; AED, automated external defibrillator; EMS, emergency medical services.

**Table 2:** Primary analysis of outcomes after out-of-hospital cardiac arrest according to base excess quartiles at hospital arrival.

	Base excess (BE), mmol/L				
	Quartile 1 (BE ≤ −21.1) n = 1528	Quartile 2 (−21.1 < BE ≤ −15.7) n = 1520	Quartile 3 (−15.7 < BE ≤ −10.4) n = 1513	Quartile 4 (BE > −10.4) n = 1505	p for trend
***Primary outcome***					
Favourable neurological survival, n (%)	49 (3.2)	72 (4.7)	149 (9.9)	356 (23.7)	<0.001
Crude OR (95 % CI)	0.11 (0.079–0.15)	0.16 (0.12–0.21)	0.35 (0.29–0.43)	Reference	
Adjusted OR (95 % CI)*	0.13 (0.090–0.19)	0.20 (0.15–0.28)	0.37 (0.29–0.48)	Reference	
***Secondary outcome***					
Survival, n (%)	101 (6.6)	174 (11.5)	271 (17.9)	475 (31.6)	<0.001
Crude OR (95 % CI)	0.15 (0.12–0.19)	0.28 (0.23–0.34)	0.47 (0.40–0.56)	Reference	
Adjusted OR (95 % CI)*	0.18 (0.14–0.24)	0.34 (0.27–0.43)	0.52 (0.42–0.63)	Reference	

Abbreviations: OR, odds ratio; CI, confidence interval; CPR, cardiopulmonary resuscitation; EMS, emergency medical services; AED, automated external defibrillator.

* Adjusted for age, sex, cause of arrest, bystander CPR, AED shock delivery by bystander, initial documented rhythm at the scene and in hospital, adrenaline administration by EMS, advanced airway management by EMS, defibrillation by EMS, no-flow time for CPR, time from witnessed arrest to hospital arrival, and time from hospital arrival to blood sampling.

A similar trend was observed for one-month survival, with the lowest proportion in the Q1 group and the highest in the Q4 group, consistent with the pattern observed for the primary outcome.

In the multivariate logistic regression analysis, the adjusted odds of a favourable neurological outcome were significantly lower in the Q1 group compared with the Q4 group (AOR, 0.13; 95 % CI, 0.090–0.19; Table 2). The sensitivity analyses incorporating either PCO_2_ and creatinine levels upon hospital arrival or low-flow time for CPR yielded similar results (Supplementary Table 2), supporting the robustness of the primary findings.

**Fig. 2:**
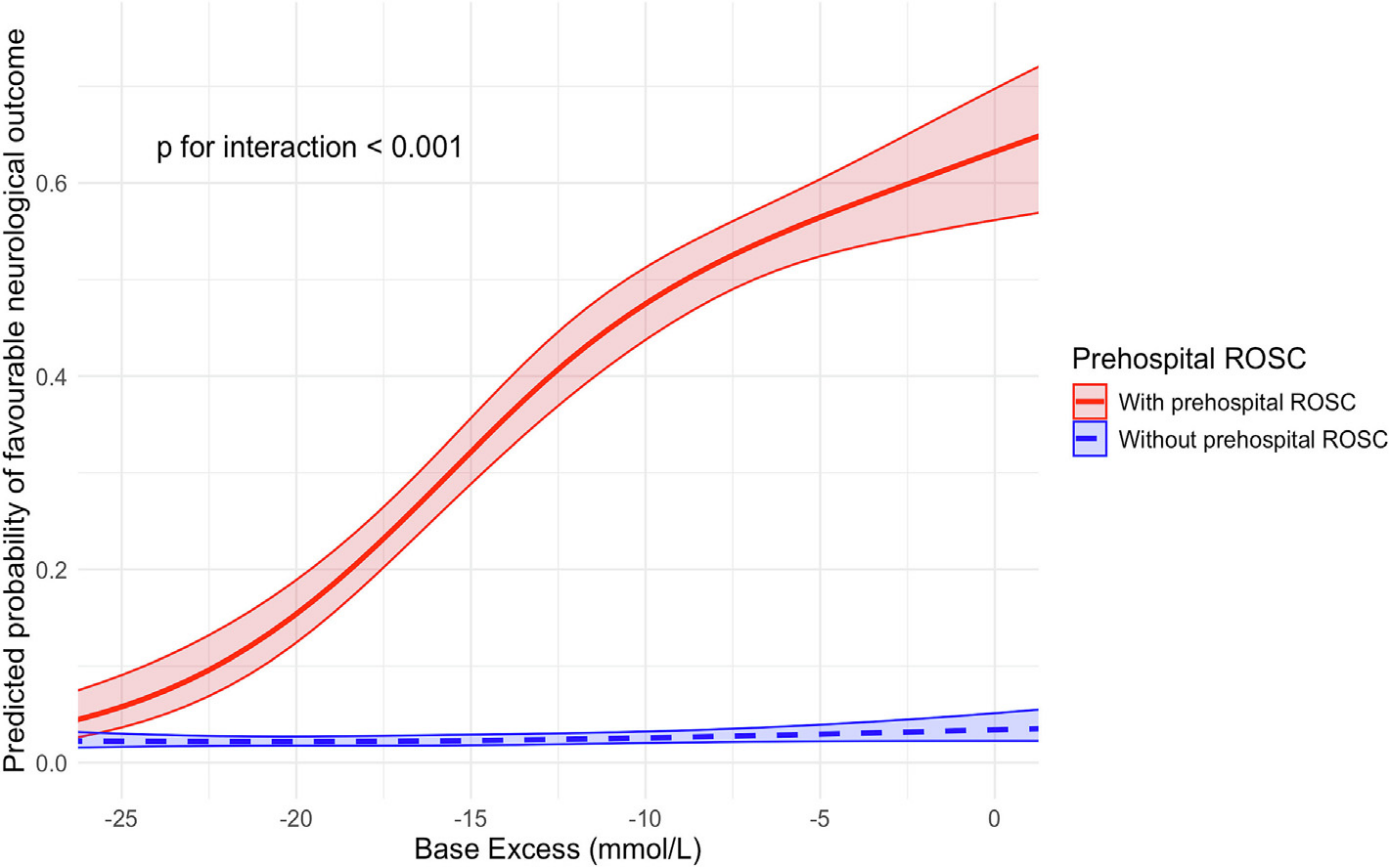
Non-linear association between base excess values and the estimated probability of favourable neurological outcomes among eligible patients, analysed using restricted cubic splines in a univariable logistic regression model stratified by subgroup. Lines indicate the estimated probabilities, whereas shaded areas represent the 95% confidence intervals. The solid red line denotes patients who achieved prehospital return of spontaneous circulation (ROSC), whereas the dotted blue line represents those who did not achieve prehospital ROSC.

Subgroup analysis revealed a significant interaction between prehospital ROSC and neurological outcome (p for interaction <0.001; Fig. 2, Table 3). Among patients who achieved prehospital ROSC, favourable neurological outcomes decreased significantly with lower BE values (p for trend <0.001; Table 3). However, no significant trend was observed among patients without prehospital ROSC (p for trend = 0.095; Table 3). A similarly strong association was observed between BE and 1-month survival in patients with prehospital ROSC.

**Table 3:** Outcomes after out-of-hospital cardiac arrest based on base excess values and prehospital ROSC status.

	Base excess (BE), mmol/L					
	Quartile 1 (BE ≤ −21.2) n = 1528	Quartile 2 (−21.2 < BE ≦ −15.7) n = 1520	Quartile 3 (−15.7 < BE ≦ −10.4) n = 1513	Quartile 4 (BE > −10.4) n = 1505	p for trend	p for interaction
***Primary outcome***						
*With prehospital ROSC* (n = 1393)						<0.001
n/N (%)	21/209 (10.1)	45/285 (15.8)	124/340 (36.5)	324/559 (58.0)	<0.001	
Crude OR (95 % CI)	0.081 (0.050–0.13)	0.14 (0.095–0.19)	0.42 (0.32–0.55)	Reference		
*Without prehospital ROSC* (n = 4673)						
n/N (%)	28/1319 (2.1)	27/1235 (2.2)	25/1173 (2.1)	32/946 (3.4)	0.095	
Crude OR (95 % CI)	0.62 (0.37–1.04)	0.64 (0.38–1.07)	0.62 (0.37–1.06)	Reference		
***Secondary outcome***						
*With prehospital ROSC* (n = 1393)						<0.001
n/N (%)	42/209 (20.1)	92/285 (32.3)	187/340 (55.0)	406/559 (72.6)	<0.001	
Crude OR (95 % CI)	0.095 (0.064–0.14)	0.18 (0.13–0.24)	0.46 (0.35–0.61)	Reference		
*Without prehospital ROSC* (n = 4673)						
n/N (%)	59/1319 (4.5)	82/1235 (6.6)	84/1173 (7.2)	69/946 (7.3)	0.0035	
Crude OR (95 % CI)	0.60 (0.42–0.85)	0.90 (0.65–1.26)	0.98 (0.70–1.36)	Reference		

Abbreviations: ROSC, return of spontaneous circulation; OR, odds ratio; CI, confidence interval.

The p-value for interaction was calculated to evaluate the effect modification between base excess values and the presence of ROSC prior to blood sampling.

## Discussion

Consistent with the results of several previous studies,^21^, ^17^, ^18^, ^19^ this large-scale, multicentre, prospective study involving >6000 adult patients with bystander-witnessed OHCA demonstrated that lower BE values upon hospital arrival were associated with poorer neurological outcomes. This association remained significant even after adjusting for the time interval from the onset of cardiac arrest to blood sampling. This finding suggests that the BE value upon hospital arrival may serve as a useful prognostic indicator of neurological outcomes after OHCA.

Corral et al. previously reported an association between lower venous BE values—measured at the initiation of CPR—and unfavourable neurological outcomes in 1552 patients with OHCA.^17^ Similarly, Matthieu et al. identified a relationship between base deficit and intensive care unit (ICU) mortality among 826 patients with OHCA admitted to the intensive care unit.^19^ However, these studies did not adjust for the interval between the occurrence of cardiac arrest and the BE measurement.^17^, ^19^ By contrast, the present study demonstrated an association between BE values upon hospital arrival and neurological outcomes in patients with OHCA, even after adjusting for the interval from cardiac arrest to BE measurement.

Subgroup analysis identified an association between the presence of prehospital ROSC and neurological outcomes. This finding may be attributed to several physiological and clinical factors.

First, the impact of cardiac arrest on tissue and organ perfusion may vary substantially depending on the ROSC status. Patients who achieved and did not achieve ROSC exhibit distinct physiological and metabolic profiles, and the trajectory of BE values may vary accordingly. In patients with prehospital ROSC, BE levels are presumed to improve over time as circulation is restored and tissue reperfusion occurs. In patients who arrive at the hospital while receiving CPR, BE may continue to decline due to persistent tissue hypoperfusion and anaerobic metabolism. Additionally, organs with high oxygen demand—such as the brain and heart—are particularly vulnerable to early oxygen deprivation, potentially resulting in localised metabolic acidosis.^32^ Under such conditions, peripheral blood samples may not accurately reflect the overall metabolic status. This discrepancy may be more pronounced in patients who do not achieve ROSC, owing to the more severe and prolonged circulatory failure compared with those who achieve ROSC. Conversely, the achievement of ROSC often indicates the provision of high-quality CPR and the timely administration of emergency interventions, such as bystander CPR or defibrillation with an AED. These measures are presumed to preserve a degree of organ perfusion, enabling BE values to more accurately reflect the true metabolic status and extent of neurological injury.

Second, a possible explanation for the lack of association between BE and neurological outcomes in patients who did not achieve ROSC is that the majority of this subgroup had already experienced poor outcomes (Fig. 2). In these patients, the prognosis may have been determined regardless of BE values, thereby limiting the utility of BE as a prognostic indicator.

Therefore, these findings suggest that the association between BE and neurological outcomes may vary according to ROSC status, with a stronger association observed among patients who achieved ROSC.^32^

One of the strengths of this study is the use of BE, rather than lactate or pH, as a prognostic marker. BE is a calculated parameter that reflects the amount of metabolic component required to restore the blood pH to normal under standardised conditions. It is derived primarily from pH and pCO_2_ levels—both of which are indicative of acid–base disturbances caused by insufficient oxygen delivery and impaired carbon dioxide clearance. Hence, BE may provide a comprehensive assessment of acid–base status compared with individual markers.^15^ Conversely, lactate is a well-established indicator of anaerobic metabolism and tissue hypoxia; however, it does not fully reflect the extent of metabolic derangement, as its levels may be influenced by hepatic clearance, renal function, and intravenous fluid administration.^10^ By contrast, BE reflects both lactate-induced acidosis and additional metabolic components, providing a more robust measure of systemic metabolic disturbance in critically ill patients.

In the present study, although the majority of patients in the lowest BE quantile (BE ≤ –21.1 mmol/L) exhibited poor neurological outcomes, a small but meaningful proportion (3.2 %) achieved a favourable outcome. This finding indicates that favourable outcomes may still be achievable even in patients with severely abnormal BE levels. Accordingly, BE should not be used in isolation to determine whether resuscitation efforts upon hospital arrival should be terminated. Instead, it should be considered as part of a broader clinical assessment that incorporates multiple prognostic factors. The development of prognostic models and clinical decision-making tools that include BE as a predictive variable may be useful for improving patient care.

This study has some limitations. First, the registry lacked detailed information on patient’s medical histories, comorbidities, and medications. The interpretation of BE values can be challenging in individuals with kidney failure, chronic pulmonary disease or complex acid–base imbalances. In a patient with chronic respiratory acidosis with a sustained increase in PCO_2_ levels by 10 mmHg over several days, the BE value may increase by approximately 4 mmol/L.^15^ During acute elevations in PCO_2_, renal compensation is limited, resulting in a minimal immediate effect on BE.^33^, ^34^ Among patients with OHCA, determining whether elevated PCO_2_ upon hospital arrival reflects a chronic condition or an acute event remains challenging. To address this uncertainty, a sensitivity analysis was performed, adjusting for PCO_2_ and creatinine levels upon hospital arrival. The findings remained consistent with those of the primary analysis (Supplementary Table 2).

Second, variations in BE measurement methods and the types of blood gas analysers used across institutions may have introduced potential inconsistencies. This registry did not distinguish between ABE and SBE, which are calculated using different formulas. To address this limitation, SBE was recalculated using arterial pH and pCO_2_ values obtained upon hospital arrival, and the primary analysis was repeated (Supplementary Table 3). The consistency of these results with the original findings supports the robustness of our findings.

Third, neurological outcomes were assessed by treating physicians using the CPC scale without blinding, which may have introduced assessment bias. However, previous studies have demonstrated good inter-rater reliability when CPC scores are categorised into favourable (CPC 1–2) and unfavourable (CPC 3–5),^35^ suggesting that any resulting bias in the primary outcome was likely minimal.

## Conclusion

This observational study of adult patients with OHCA demonstrated that lower BE values upon hospital arrival were associated with poorer neurological outcomes. BE may serve as a useful prognostic indicator, particularly in patients with prehospital ROSC.

## Funding sources

This study was supported by a scientific research grant from the 10.13039/501100001691JSPS KAKENHI of Japan (22H03313 to Iwami, 22K09139 to Kitamura and 22K2144 to Nishioka).

## CRediT authorship contribution statement

**Ryuta Onodera:** Writing – original draft, Visualization, Software, Project administration, Methodology, Formal analysis, Data curation, Conceptualization. **Norihiro Nishioka:** Writing – review & editing, Writing – original draft, Validation, Supervision, Project administration, Methodology, Funding acquisition, Conceptualization. **Tomoki Yamada:** Resources, Investigation. **Shunichiro Nakao:** Resources, Investigation. **Kazuhisa Yoshiya:** Resources, Investigation. **Changhwi Park:** Resources, Investigation. **Tetsuro Nishimura:** Resources, Investigation. **Takuya Ishibe:** Resources, Investigation. **Kazuma Yamakawa:** Resources, Investigation. **Takeyuki Kiguchi:** Resources, Investigation. **Masafumi Kishimoto:** Resources, Investigation. **Kohei Ninomiya:** Resources, Investigation. **Yusuke Ito:** Resources, Investigation. **Taku Sogabe:** Resources, Investigation. **Takaya Morooka:** Resources, Investigation. **Haruko Sakamoto:** Resources, Investigation. **Yuki Hironaka:** Resources, Investigation. **Atsunori Onoe:** Resources, Investigation. **Tasuku Matsuyama:** Resources, Data curation. **Yohei Okada:** Writing – review & editing, Resources, Data curation. **Satoshi Matsui:** Resources, Data curation. **Satoshi Yoshimura:** Resources, Data curation. **Shunsuke Kimata:** Resources, Data curation. **Shunsuke Kawai:** Resources, Data curation. **Yuto Makino:** Writing – review & editing, Resources, Data curation. **Ling Zha:** Resources, Data curation. **Kosuke Kiyohara:** Resources, Data curation. **Tetsuhisa Kitamura:** Resources, Funding acquisition, Data curation. **Taku Iwami:** Writing – review & editing, Supervision, Project administration, Methodology, Funding acquisition.

## Declaration of competing interest

YO received a research grant from the Zoll Foundation. The other authors declare that they have no recognised financial or personal conflicts of interest that could have influenced the work presented in this study.
