# Supplementary material for: Association between base excess level at hospital arrival and neurological outcomes in adult out-of-hospital cardiac arrest: A multicentre cohort study
Source: Resusc Plus. 2025 Aug 6;25:101055. doi: 10.1016/j.resplu.2025.101055 (PMC12447215; doi:10.1016/j.resplu.2025.101055)
Supplement: Supplementary Data 1 [file mmc1.docx]

**Supplementary Table 1. Summary of missing data**

| Variables | Total  n = 6,066 |
| --- | --- |
|  |  |
| ***Prehospital characteristics*** |  |
| Age | 0 (0%) |
| Male | 0 (0%) |
| Cardiac cause | 0 (0%) |
| Type of cardiac cause | 0 (0%) |
| Layperson bystander CPR | 0 (0%) |
| Type of bystander | 0 (0%) |
| Bystander shock delivery by an AED | 0 (0%) |
| Initial documented rhythm | 0 (0%) |
| Defibrillation by EMS | 0 (0%) |
| Adrenaline administration by EMS | 0 (0%) |
| Advanced airway management by EMS | 0 (0%) |
| No-flow time for CPR | 188 (3.1%) |
| Low-flow time for CPR | 232 (3.8%) |
| Time from witness to hospital arrival | 48 (0.8%) |
| Time from call to hospital arrival | 52 (0.9%) |
| ***In-hospital characteristics*** |  |
| Initial documented rhythm | 0 (0%) |
| Defibrillation in hospital | 0 (0%) |
| Time from hospital arrival to blood test | 0 (0%) |
| Coronary angiography | 0 (0%) |
| Target temperature management | 0 (0%) |
| Extracorporeal life support initiated after blood sampling | 0 (0%) |
| pH | 6 (0.1%) |
| Lactate | 71 (1.2%) |
| HCO_3_^–^ | 29 (0.5%) |
| PaCO_2_ | 1 (0.02%) |
| Creatinine | 1,613 (26.6%) |
| ***Outcome*** |  |
| Favourable neurological survival | 0 (0%) |
| Survival | 0 (0%) |

Numbers and percentages (%). CPR, cardiopulmonary resuscitation; AED, automated external defibrillator; EMS, emergency medical services

**Supplementary Table 2. Sensitivity analysis of the outcomes after out-of-hospital cardiac arrest according to base excess values, adjusted for either arterial carbon dioxide and creatinine levels upon hospital arrival or low-flow time for cardiopulmonary resuscitation**

|  | Base excess (BE), mmol/L | | | | |
| --- | --- | --- | --- | --- | --- |
|  | Quartile 1  (BE $\leq$ −21.1)  n = 1,528 | Quartile 2  (−21.1< BE ≤ −15.7)  n = 1,520 | Quartile 3  (−15.7 < BE ≤ −10.4)  n = 1,513 | Quartile 4  (BE > −10.4)  n = 1,505 | p for trend |
| ***Primary outcome*** |  |  |  |  |  |
| Favourable neurological survival, n (%) | 49 (3.2) | 72 (4.7) | 149 (9.9) | 356 (23.7) | <0.001 |
| Crude OR (95% CI) | 0.11 (0.079–0.15) | 0.16 (0.12–0.21) | 0.35 (0.29–0.43) | Reference |  |
| Adjusted OR (95% CI)† | 0.17 (0.11–0.26) | 0.26 (0.18–0.37) | 0.44 (0.33–0.60) | Reference |  |
| Adjusted OR (95% CI)‡ | 0.33 (0.22–0.49) | 0.38 (0.27–0.54) | 0.56 (0.42–0.75) | Reference |  |
| ***Secondary outcome*** |  |  |  |  |  |
| Survival, n (%) | 101 (6.6) | 174 (11.5) | 271 (17.9) | 475 (31.6) | <0.001 |
| Crude OR (95% CI) | 0.15 (0.12–0.19) | 0.28 (0.23–0.34) | 0.47 (0.40–0.56) | Reference |  |
| Adjusted OR (95% CI)† | 0.21 (0.16–0.28) | 0.40 (0.31–0.51) | 0.58 (0.46–0.73) | Reference |  |
| Adjusted OR (95% CI)‡ | 0.36 (0.27–0.48) | 0.56 (0.44–0.71) | 0.73 (0.58–0.91) | Reference |  |

Abbreviations: OR, odds ratio; CI, confidence interval

†Partial pressure of arterial carbon dioxide and creatinine levels upon hospital arrival were included in the primary analysis model.

‡Low-flow time for cardiopulmonary resuscitation was included in the primary analysis model.

**Supplementary Table 3. Sensitivity analysis of the outcomes after out-of-hospital cardiac arrest according to the recalculated standard base excess values**

|  | Standard base excess (SBE), mmol/L | | | | |
| --- | --- | --- | --- | --- | --- |
|  | Quartile 1  (BE $\leq$ −20.5)  n = 1,515 | Quartile 2  (−20.5 < BE ≤ −15.2)  n = 1,515 | Quartile 3  (−15.2 < BE ≤ −10.0)  n = 1,515 | Quartile 4  (BE > −10.0)  n = 1,514 | p for trend |
| ***Primary outcome*** |  |  |  |  |  |
| Favourable neurological survival, n (%) | 51 (3.4) | 90 (5.9) | 166 (11.0) | 318 (21.0) | <0.001 |
| Crude OR (95% CI) | 0.13 (0.097–0.18) | 0.24 (0.19–0.30) | 0.46 (0.38–0.57) | Reference |  |
| Adjusted OR (95% CI)^*^ | 0.21 (0.15–0.30) | 0.29 (0.21–0.39) | 0.48 (0.37–0.62) | Reference |  |
| ***Secondary outcome*** |  |  |  |  |  |
| Survival, n (%) | 99 (6.5) | 191 (12.6) | 290 (19.1) | 439 (29.0) | <0.001 |
| Crude OR (95% CI) | 0.17 (0.14–0.22) | 0.35 (0.29–0.43) | 0.58 (0.49–0.69) | Reference |  |
| Adjusted OR (95% CI)^*^ | 0.23 (0.18–0.30) | 0.41 (0.33–0.51) | 0.61 (0.50–0.75) | Reference |  |

Abbreviations: OR, odds ratio; CI, confidence interval; CPR, cardiopulmonary resuscitation; EMS, emergency medical services; AED, automated external defibrillator

^*^Adjusted for age, sex, cause of arrest, bystander CPR, AED shock delivery by bystander, initial documented rhythm at the scene and in the hospital, adrenaline administration by EMS, advanced airway management by EMS, defibrillation by EMS, no-flow time for CPR, time from witnessed cardiac arrest to hospital arrival, and time from hospital arrival to blood sampling

**Supplementary Figure 1. Nonlinear relationship between base excess values and the estimated probability of a favourable neurological outcome
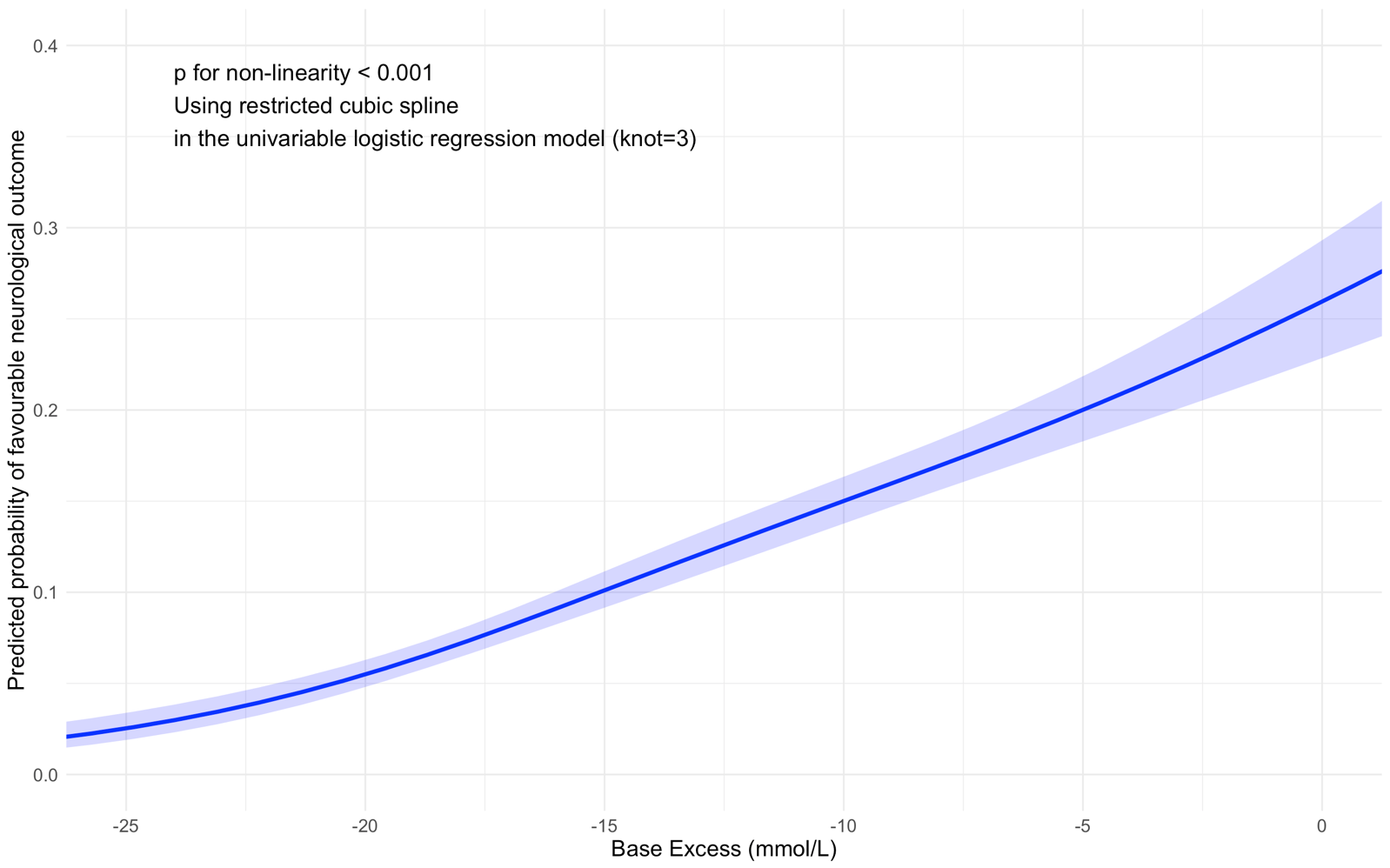
**

The lines indicate estimated the probabilities, whereas the shaded areas represent the 95% confidence intervals.
